# Growth in height and its association with overweight and obesity in Mexican children: an evaluation based on a nationally representative sample (ENSANUT 2018)

**DOI:** 10.3389/fpubh.2024.1339195

**Published:** 2024-03-08

**Authors:** Luis Alberto Flores, Sudip Datta Banik, Noel Cameron, Isabel Januário Fragoso

**Affiliations:** ^1^Autonomous University of Chihuahua, Chihuahua, Mexico; ^2^Center for Research and Advanced Studies - Mérida Unit, Merida, Mexico; ^3^School of Sport, Exercise and Health Sciences, Loughborough University, Loughborough, United Kingdom; ^4^CIPER, Faculdade de Motricidade Humana, Universidade de Lisboa, Cruz Quebrada, Portugal

**Keywords:** peak height velocity, growth velocity, adolescence, somatic maturity, children

## Abstract

The present study aimed to estimate the height growth curve for Mexican boys and girls based on their body mass index (BMI) status (normal and overweight/obese) and to develop a height Lambda, Mu, and Sigma (LMS) growth reference for Mexican children aged 2 to 18 years.

**Methods:**

Chronological age and height records (7,097 boys and 6,167 girls) were obtained from the Mexican National Survey of Health and Nutrition database. Height growth curves were fitted using the Preece-Baines 1 (PB1) model and the LMS method.

**Results:**

Age at peak height velocity (APHV) was 12.4 and 12.7 years for overweight-obese and normal-weight boys, respectively, and was 9.6 and 10.4 years for overweight-obese and normal-weight girls, respectively. Growth velocity was higher at the age of take-off (TO) in overweight-obese children than in normal-weight children (5.2 cm/year vs. 5 cm/year in boys and 6.1 cm/year vs. 5.6 cm/year in girls); nevertheless, the growth velocity at APHV was higher for normal-weight children than for overweight-obese children (7.4 cm/year vs. 6.6 cm/year in boys and 6.8 cm/year vs. 6.6 cm/year in girls, respectively). Distance curves developed in the present study and by the World Health Organization (WHO) using LMS showed similar values for L and S parameters and a higher M value compared with the WHO reference values.

**Conclusion:**

This study concluded that overweight-obese children had earlier APHV and lower PHV than normal-weight children. Furthermore, Mexican children and adolescents were shorter than the WHO growth reference by age and sex.

## Introduction

Human growth and development, which occur during the first 18 years of life, are biological phenomena where changes in body dimensions (absolute and relative to height) and composition occur until final maturation (1). The modeling of a child’s growth curve and the analysis of the timing and tempo in which different growth and maturation events occur are topics of great interest in various fields, such as health sciences, nutrition, biological anthropology, and sports, where growth plays a crucial role in explaining or confounding factors. Monitoring child growth and development is also important to enable the comparison of individual physical growth with reference population trends (by age and sex) to analyze growth deficits, if any, and nutritional status, which is one of the major public health concerns of any country (2).

One of the most important maturity indicators is the age of occurrence and magnitude of peak height velocity (PHV), which refers to the time at which the maximum increase in height is achieved during adolescence (3), in which, significant physiological changes occur, including the development of secondary sexual characteristics and body composition changes. PHV typically occurs between the ages of 9 and 15 years, although the exact age can vary. The monitoring of PHV is crucial for evaluating the attainment of an individual’s maturity and providing insights into the timing of growth-related milestones (3).

There is a relative scarcity of publications on child growth and modeling of the Mexican population compared to other countries and continents (Americas and Europe). Recently, differential timing and tempo of the height growth of children and adolescents aged 9 to 17 years from Merida City, Mexico, were analyzed using the Preece-Baines 1 (PB1) model. The authors of this study reported an age at PHV (APHV) of 11 years in girls and 12.4 years in boys (3). Other studies conducted on the Mexican population were related to secular changes in height (4). Malina et al. (5) reported secular changes in height and sitting height in children and adolescents (between 1978 and 2000) from a rural community in Oaxaca, Mexico. This study used data from Malina et al. (6), Faulhaber and Villanueva (7), Faulhaber (8), and Peña-Reyes et al.’s studies (9) of Mexican children and adolescents.

Differential height growth and velocity patterns of normal-weight and overweight-obese children and adolescents have been established in the United States and Europe (10–13). Overweight-obese children are taller for their age during childhood but have a lower relative height growth during adolescence, leading to shorter heights in adulthood compared to normal-weight peers; moreover, overweight children are taller as they mature earlier than their peers who are lean (14). Overweight-obese children experience earlier APHV (from 1 to 3 years) and greater PHV compared to their peers who are lean (15). Childhood obesity is related to early puberty and menarche (16). Hypotheses suggest that adiposity, increased leptin, and sex hormones in obese children accelerate pubertal growth (11, 13). Elevated leptin levels and the leptin-to-adiponectin ratio predict obesity gain in non-obese, prepubertal children (17). Pre-pubertal weight gain is associated with earlier maturity, advanced bone age, and reduced GH secretion (14, 18, 19). However, it remains unclear whether early maturation causes weight gain or if elevated body mass index (BMI) and body fatness induce an early maturation (20).

This topic is relevant because there is a high prevalence of childhood overweight and obesity in Mexico. Pérez-Herrera et al. (21) reported an overweight and obesity prevalence of 35.6% in children aged 5 to 11 years (18.1 and 17.5% of overweight and obese children, respectively) and 38.4% among adolescents aged 12 to 19 years (23.8 and 14.6% of overweight and obese adolescents, respectively), which has been increasing in recent years (18). However, the prevalence of chronic undernutrition (low length/height-for-age or stunting) among Mexican children and adolescents is also remarkable. In 2016, 10% of the population under 5 years were stunted (22). The coexistence of both extremes of malnutrition, stunting, and overweight in Mexican children, which is defined as the double burden of malnutrition, has been reported in Mérida, Yucatán (23), and some other states (24). Socioeconomic inequalities in Mexican society are the main factors behind children’s malnutrition and growth deficits (25). In 2018, particularly in rural and suburban areas of Mexico, 22.6 and 32.9% of the population presented moderate-to-severe and low household food insecurity, respectively (26).

Taking into account that growth curves are the best indicator of the growth pattern of children and adolescents in a given population (27) and that in the Mexican population, there coexist two extreme situations of malnutrition, overweight and stunting (23, 24), and the purpose of the present study is to clarify the impact of overweight on growth and maturation patterns, using the development of mathematical growth curves when a representative growth reference curve for the Mexican population is not available.

Therefore, the objectives of the present study, using a nationally representative database, are as follows: (1) to estimate height growth velocity (of normal and overweight) of boys and girls by age, using the PB 1 model to understand whether overweight and normal-weight children and adolescents, as previously reported, have different growth patterns and (2) to develop normalized height growth curves for boys aged 2 to 18 years and girls aged 2 to 16 years using the LMS method and compare the results with the World Health Organization (WHO) growth standards and reference values to evaluate growth patterns by age and sex, if any.

## Participants and methods

The present study used data from Mexico’s National Survey of Health and Nutrition 2018 database (ENSANUT 2018, Spanish acronym), carried out by the National Institute of Statistics and Geography (INEGI, Spanish acronym) and the National Institute of Public Health (INSP, Spanish acronym) of the federal government. The use of the database is subject to strict terms and conditions.^1^ The sampling design in the ENSANUT was multistage stratified random sampling for 32 Mexican states, and the sample obtained was probabilistic and representative of the country’s population below 19 years of age.

The height and weight data of 7,097 boys (from 1.50 to 18.49 years of age) and 6,167 girls (from 1.50 to 16.49 years of age) were recorded in ENSANUT 2018. Subsequently, the evaluation of growth and nutritional status (height-for-age, BMI) followed the protocol of the WHO child growth standards and reference charts (28–30). In the case of girls, we considered only up to 16.49 years of age, because, in our previous study, the height recorded in the age groups of 17 and 18 years was similar to the height recorded at the age of 16 years; generally, the average height growth ended at 16 years of age.

Individuals with the estimated height-for-age *z*-scores of >3 and < −3 standard deviations were eliminated from the present study, and the final sample sizes for boys and girls were 7,097 and 6,167, respectively.

The height and weight were measured following the standard protocols established by Lohman et al. (31) and Habicht (32) (explained in the ENSANUT 2018 report). Age groups were based on the rounded decimal age (i.e., 6 years: decimal age between 5.5 and 6.49 years).

### Statistical analyses

The z-test for comparison of proportions was used to identify the differences in the distribution of overweight and obesity among the different regions of Mexico, by sex.

The Preece-Baines 1 model (33) was used to estimate height growth, which was based on five parameters. All analyses were performed with the Gauss–Newton method of the statistical analysis software SAS version 9.0. In addition, age (years) at take-off (TO) (which represents the onset of adolescent growth spurt), height (cm) at take-off, velocity (cm/year) at take-off, height increase (cm) from age at TO to APHV, age (years) at PHV, height (cm) at PHV, velocity (cm/year) at PHV, final height, and increase in height from PHV to adult height were estimated. The precision of the PB1 model fitting was evaluated by the residual standard deviation (RSD), where a value less than 0.5 cm was commonly accepted to indicate a good fit (34). The PB1 models were fitted for every individual in the entire sample and separately for normal, overweight, and obese boys and girls (in underweight children, the growth curve could not be fitted due to the low sample size). Student’s *t*-test for independent samples was used to compare the estimated mathematical and biological parameters of the PB1 model between children with normal weight and children with overweight/obese, by sex.

The smoothed (normalized) percentile curves (the 3rd, 10th, 25th, 50th, 75th, 90th, and 97th centiles) for height were developed by the LMS method (35–37), using the LMS Chart Maker Pro version 2.5 by three smooth curves: Lambda (L) or power transformation, Mu (M) or mean, and Sigma (S) or the coefficient of variation. The equivalent (or effective) degrees of freedom (e.d.f.) to fit the height curves were three for L, six for M, and three for S, in both sexes. Student’s *t*-test was used to compare the height values of the Mexican population with the WHO reference values by age and sex.

The Preece-Baines 1 model parameters were used for comparison between the different BMI groups, and the LMS curves were used to characterize the pattern of growth in comparison with the WHO references and standards. The results were considered significant at the *p* < 0.05 level.

## Results

The mean values of height by age groups in boys and girls are presented in Table 1. The prevalence of overweight-obese children in the present sample was 22.5% for boys and 21% for girls in the north of Mexico, while in the central province of the country, it was 38.5 and 41.1%, and in the south, 39 and 37.9%, respectively. Differences were found in the proportion of overweight and obesity between the northern and central regions in both sexes (in boys ~0.160, CI 0.120, 0.201, *p* = 0.01; in girls ~0.200, CI 0.160, 0.240, *p* < 0.001) and between the northern and southern regions (in boys ~0.165, CI 0.125, 0.205, *p* = 0.005; in girls ~0.169, CI 0.129, 0.209, *p* = 0.01). No significant differences were found between the central and southern regions in both sexes (*p* = 0.200).

**Table 1:** Descriptive statistics of height (cm) and overweight-obese prevalence by age and sex in Mexican children and adolescents.

Age (years) and n (girls/boys)	Girls (*n* = 6,167)		Boys (*n* = 7,097)	
	Height	Overweight-obese prevalence	Height	Overweight-obese prevalence
	Mean ± SD (cm)		Mean ± SD (cm)	
2 (*n* = 385/435)	82.92 ± 4.67	7%	84.25 ± 4.67	9%
3 (*n* = 394/475)	91.60 ± 4.51	6%	93.03 ± 4.52	9%
4 (*n* = 422/452)	98.89 ± 4.82	10%	99.85 ± 4.76	8%
5 (*n* = 427/427)	105.44 ± 5.35	17%	106.64 ± 5.07	19%
6 (*n* = 434/448)	111.76 ± 5.17	25%	113.34 ± 5.65	35%
7 (*n* = 472/496)	118.43 ± 5.90	32%	119.00 ± 5.81	30%
8 (*n* = 472/446)	123.88 ± 6.01	30%	124.77 ± 6.43	38%
9 (*n* = 491/478)	129.82 ± 6.93	40%	130.63 ± 6.85	44%
10 (*n* = 450/438)	136.47 ± 7.65	40%	135.46 ± 6.69	45%
11 (*n* = 401/387)	143.23 ± 7.72	39%	141.22 ± 7.41	47%
12 (*n* = 373/390)	149.25 ± 6.77	50%	147.20 ± 8.56	48%
13 (*n* = 370/388)	152.13 ± 6.36	45%	153.62 ± 8.72	44%
14 (*n* = 336/382)	153.96 ± 6.40	43%	161.03 ± 8.25	39%
15 (*n* = 380/368)	155.87 ± 6.92	41%	164.62 ± 7.59	38%
16 (*n* = 333/376)	156.35 ± 6.87	40%	167.34 ± 6.89	38%
17 (*n* = 341)			168.38 ± 6.63	35%
18 (*n* = 370)			169.12 ± 7.24	33%

The PB1 model applied to the data of 2–18-year-old boys (*n* = 7,097) and 2–16-year-old girls (*n* = 6,167) was adequately adjusted, obtaining an *R*^2^ of 0.99 in both sexes and an RSD of 0.42 cm in girls and 0.47 cm in boys. When the PB1 model fitting was based on the nutritional status, the curve of children with adequate weight-for-age was slightly better adjusted, resulting in an RSD of less than 0.37 cm in both sexes, while, in the overweight or obese sample, the RSD was 0.72 cm in girls and 0.87 cm in boys. Table 2 shows the values of the five parameters of the PB1 model estimated using the entire sample and by the nutritional status (normal-weight and overweight-obese) of boys and girls. Significant differences were found between normal-weight and overweight-obese in both sexes in all mathematical parameters such as theta (*p* = 0.001 for boys and girls), h1 (*p* = 0.001 in boys and 0.033 in girls), h0 (*p* = 0.001 for boys and girls), s0 (*p* = 0.001 for boys and girls), and s1 (*p* = 0.001 for boys and girls).

**Table 2:** The Preece-Baines 1 (PB1) model parameters of the height growth in Mexican boys from 2 to 18 years and girls from 2 to 16 years.

Model parameters								
Parameters	Girls (n = 6,167)				Boys (n = 7,097)			
	Estimate	95% CI		RSD	Estimate	95% CI		RSD
		Lower	Upper			Lower	Upper	
*All children*								
h0 (cm)	156.6	156.0	157.1	0.417	169.7	169.1	170.3	0.468
h1 (cm)	144.2	143.6	144.8		156.5	156.0	157.0	
S0 (cm/year)	0.12	0.11	0.12		0.10	0.10	0.11	
S1 (cm/year)	0.85	0.78	0.92		0.86	0.79	0.92	
Theta (years)	11.2	11.0	11.3		13.3	13.2	13.5	
*Normal-weight children*								
h0 (cm)	156.4	155.8	157.0	0.347	168.9	168.3	169.5	0.366
h1 (cm)	144.2	143.6	144.8		155.4	154.9	155.9	
S0 (cm/year)	0.12	0.11	0.12		0.10	0.10	0.10	
S1 (cm/year)	0.89	0.81	0.97		0.94	0.86	1.01	
Theta (years)	11.4	11.2	11.5		13.4	13.2	13.5	
*Overweight-obese children*								
h1 (cm)	156.5	155.6	157.4	0.716	170.2	169.2	171.2	0.873
h0 (cm)	144.7	143.4	146.1		158.3	157.4	159.2	
S0 (cm/year)	0.13	0.11	0.14		0.11	0.11	0.12	
S1 (cm/year)	0.85	0.70	0.99		0.91	0.78	1.03	
Theta (years)	10.9	10.6	11.2		13.3	13.1	13.6	

CI, confidence intervals; h1, adult height; h0, height at peak height velocity; S0, velocity constant of prepubertal growth; S1, velocity constant of pubertal growth; Theta ( θ) , age at peak height velocity; RSD, residual standard deviation. Significant differences were found in all mathematical parameters between normal-weight and overweight-obese children in both sexes (*p* < 0.05).

Boys reached an average adult height of 169.7 cm, while girls reached an average adult height of 156.6 cm. These values are represented by the parameter h1, which is the only mathematical parameter with biological interpretation.

Table 3 shows all the biological parameters estimated by the first derivative of the PB1 model, by sex and nutritional status. Age at TO and PHV values were 8.6 and 12.4 years for boys, respectively, and 7.0 and 9.9 years for girls, respectively. The percentage of adult height achieved at PHV was 89% for boys and 87% for girls.

**Table 3:** Biological parameters estimated from the Preece-Baines 1 model of the height growth in Mexican girls from 2 to 16 years and boys from 2 to 18 years.

Biological parameters	Girls				Boys			
	All sample (*n* = 6,167)^a^	Normal-weight (*n* = 4,260)	Overweight-obese (*n* = 1880)	*p*-value	All sample (*n* = 7,097)^a^	Normal-weight (*n* = 4,739)	Overweight-obese (*n* = 2,254)	*p*-value
Age (years) at PHV	9.94	10.37	9.47	**0.001**	12.41	12.67	12.40	**0.001**
Age (years) at TO	7.00	7.17	7.01	**0.030**	8.65	8.72	9.00	**0.016**
Height (cm) at PHV	136.28	137.47	135.78	0.169	150.31	150.37	152.37	0.155
Height (cm) at TO	117.74	117.86	120.23	0.061	127.97	126.68	132.56	**0.001**
Velocity (cm/year) at PHV	6.75	6.79	6.60	**0.011**	6.87	7.44	6.62	**0.001**
Velocity (cm/year) at TO	5.93	5.62	6.12	**0.001**	5.24	4.95	5.21	**0.001**
Increase of stature from TO to PHV (cm)	18.54	19.61	15.55	**0.001**	22.34	23.69	19.81	**0.001**
Increase (cm) of stature from PHV to adult stature	20.32	18.93	20.72	**0.001**	19.39	18.53	17.83	**0.010**
Intensity TO	0.82	1.18	0.48	**0.001**	1.63	2.49	1.41	**0.001**

PHV, peak height velocity; TO, take-off.

a 27 girls and 104 boys were classified as “underweight” according to the WHO, so they were used in the modeling of the growth curve in “all sample” but were discarded when plotting growth in “normal weight” and “overweight-obese”. The bold values represent the statistically significant *p*-values.

The results of the PB1 model fitted in the samples by nutritional status (normal-weight and overweight-obese) showed age at PHV was earlier for children with overweight-obese compared to the normal weight-for-age peers, 12.4 years vs. 12.7 years among boys (*t* = 4.77, *p* = 0.001) and 9.5 years vs. 10.4 years among girls (*t* = 32.11, *p* = 0.001), respectively. Growth velocity (cm/year) at the age of TO was higher in overweight-obese individuals than in the children of adequate weight (5.2 cm/year vs. 4.9 cm/year, *t* = 3.88, *p* = 0.001, in boys, and 6.1 cm/year vs. 5.6 cm/year, t = 6.56, *p* = 0.001, in girls). However, growth velocity at the age of PHV was higher for normal-weight boys than for overweight-obese boys (7.4 cm/year vs. 6.6 cm/year, t = 9.27, *p* = 0.001) and girls (6.8 cm/year vs. 6.6 cm/year, *t* = 2.31, *p* = 0.011) (Table 3). No differences were found in height at PHV between normal-weight and overweight-obese children in both sexes (*t* = 1.02, *p* = 0.155 in boys and *t* = 0.96, *p* = 0.169 in girls).

**Figure 1:**
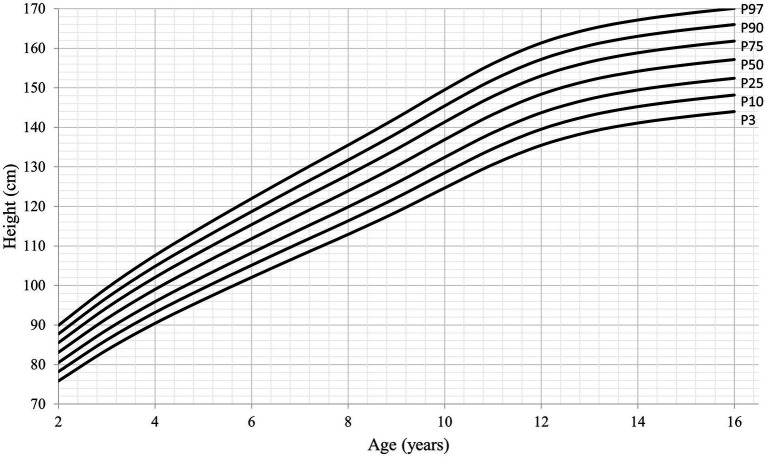
Height-for-age percentiles for girls (2–16 years).

**Figure 2:**
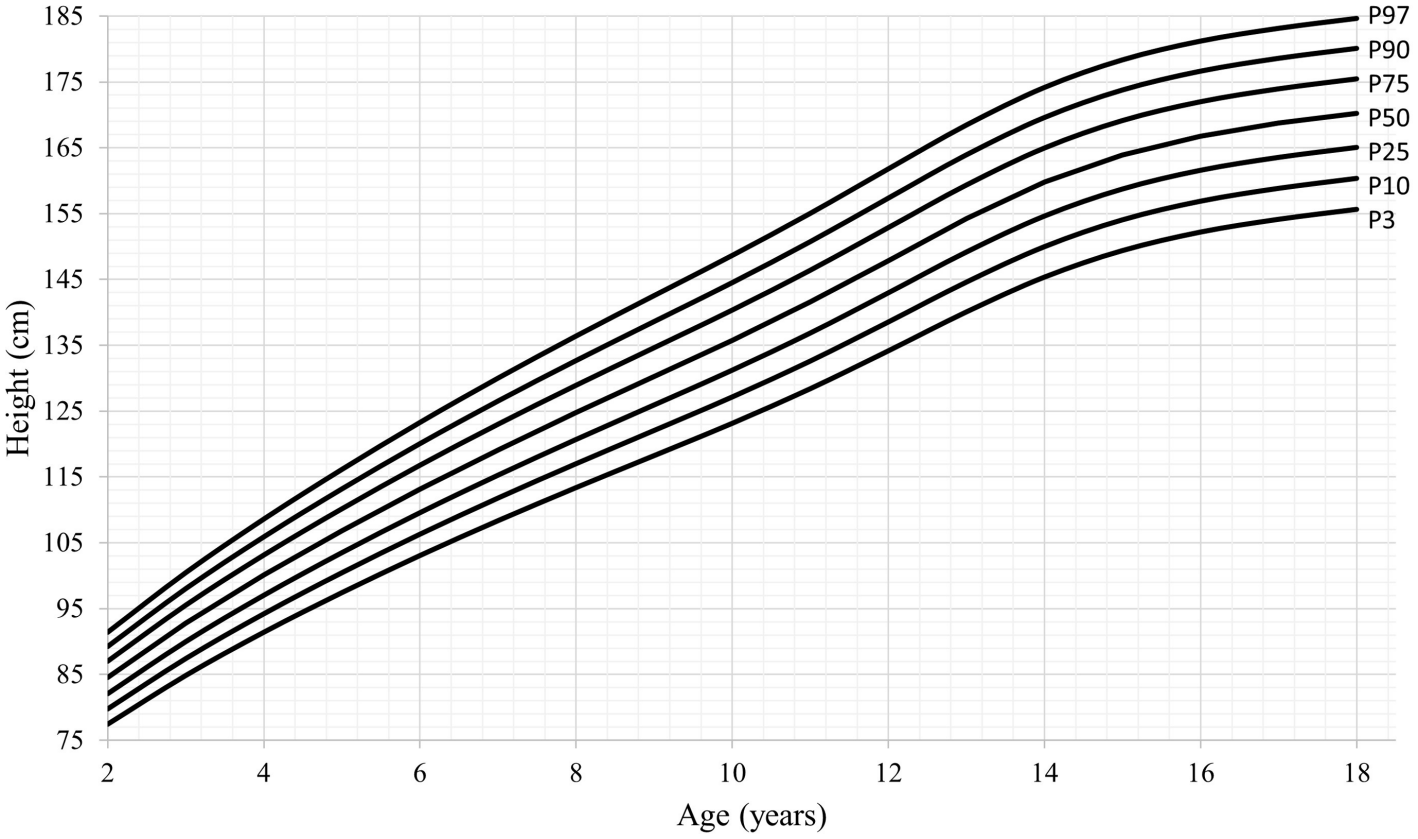
Height-for-age percentiles for boys (2–18 years).

Table 4 shows the corresponding values of the height growth curves smoothed by LMS, observed in the present study, and the WHO growth standards and reference values, by age and sex. The coefficient of variation (S value) ranged between 4.6 and 4.9% in girls and 4.5 and 5.0% in boys, similar to what is presented in the WHO growth standards and reference values. With respect to the L parameter, the highest values were observed at the age of 2–5 years (1.07–1.39) and 14–18 years (1.04–1.19) in boys, while corresponding values among girls were at the age of 2–3 years (1.55 and 1.20, respectively) and 13–16 years (1.02–1.17); furthermore, slightly higher values were observed at these ages compared to the WHO reference values (Table 4). When comparing the height values of the Mexican population with the reference values of the WHO, significant differences were found in all ages of both sexes (*p* = 0.001). Height growth curves for the 3rd, 10th, 25th, 50th, 75th, 90th, and 97th percentiles for 2 to 18-year-old boys and 2 to 16-year-old girls are presented in Figures 1, 2, respectively.

**Table 4:** LMS and percentiles values of height for Mexican children.

	Study	Girls (*n* = 6,167)										Boys (*n* = 7,097)									
Age		L	M	S	P3	P10	P25	P50	P75	P90	P97	L	M	S	P_3_	P_10_	P_25_	P_50_	P_75_	P_90_	P_97_
2	PS	1.55	83.04	0.04	75.9	78.2	80.5	83.0	85.5	87.7	89.9	1.39	84.55	0.04	77.5	79.8	82.0	84.6	87.0	89.2	91.4
	WHO	1.00	90.68	0.04	84.0	86.2	88.3	90.7	93.1	95.2	97.3	1.00	91.93	0.04	85.5	87.6	89.6	91.9	94.2	96.3	98.3
3	PS	1.20	91.56	0.05	83.7	86.2	88.7	91.6	94.4	96.9	99.3	1.28	92.76	0.04	84.9	87.4	90.0	92.8	95.5	98.0	100.5
	WHO	1.00	99.04	0.04	91.4	93.8	96.3	99.0	101.8	104.3	106.7	1.00	99.85	0.04	92.4	94.8	97.2	99.9	102.5	104.9	107.3
4	PS	0.90	98.97	0.05	90.4	93.1	95.9	99.0	102.0	104.8	107.6	1.18	100.08	0.05	91.4	94.2	97.0	100.1	103.2	105.9	108.6
	WHO	1.00	106.17	0.04	97.6	100.4	103.1	106.2	109.2	112.0	114.7	1.00	106.67	0.04	98.4	101.0	103.7	106.7	109.6	112.3	115.0
5	PS	0.67	105.53	0.05	96.4	99.3	102.2	105.5	108.9	111.9	114.9	1.07	106.82	0.05	97.4	100.4	103.5	106.8	110.2	113.2	116.1
	WHO	1.00	112.18	0.04	102.9	105.6	108.8	112.2	115.5	118.8	121.5	1.00	112.91	0.04	104.0	106.6	109.7	112.9	116.1	119.3	121.8
6	PS	0.49	111.79	0.05	102.1	105.1	108.2	111.8	115.4	118.7	122.0	0.96	113.16	0.05	103.1	106.3	109.5	113.2	116.8	120.1	123.3
	WHO	1.00	117.98	0.04	108.0	110.9	114.4	118.0	121.5	125.1	127.9	1.00	118.87	0.04	109.3	112.1	115.4	118.9	122.3	125.8	128.5
7	PS	0.38	117.88	0.05	107.5	110.8	114.1	117.9	121.7	125.3	128.8	0.86	119.12	0.05	108.4	111.8	115.2	119.1	123.0	126.5	130.0
	WHO	1.00	123.66	0.05	113.1	116.1	119.9	123.7	127.5	131.2	134.3	1.00	124.54	0.04	114.3	117.2	120.8	124.5	128.2	131.9	134.8
8	PS	0.36	123.87	0.05	112.9	116.3	119.9	123.9	128.0	131.7	135.5	0.79	124.78	0.05	113.4	117.0	120.7	124.8	128.9	132.7	136.4
	WHO	1.00	129.50	0.05	118.3	121.5	125.5	129.5	133.5	137.5	140.7	1.00	129.93	0.04	119.0	122.1	126.0	129.9	133.9	137.8	140.9
9	PS	0.44	130.14	0.05	118.6	122.2	125.9	130.1	134.4	138.4	142.3	0.75	130.25	0.05	118.2	122.0	125.9	130.3	134.6	138.6	142.5
	WHO	1.00	135.54	0.05	123.8	127.2	131.3	135.5	139.8	143.9	147.3	1.00	135.18	0.05	123.5	126.9	131.0	135.2	139.4	143.5	146.8
10	PS	0.61	136.85	0.05	124.7	128.5	132.4	136.9	141.3	145.4	149.5	0.76	135.74	0.05	123.1	127.1	131.2	135.7	140.3	144.5	148.6
	WHO	1.00	141.79	0.05	129.5	133.1	137.4	141.8	146.2	150.6	154.1	1.00	140.39	0.05	128.1	131.6	136.0	140.4	144.8	149.2	152.7
11	PS	0.79	143.23	0.05	130.6	134.6	138.7	143.2	147.8	152.0	156.1	0.80	141.59	0.05	128.4	132.6	136.8	141.6	146.4	150.7	155.0
	WHO	1.00	148.18	0.05	135.5	139.2	143.6	148.2	152.7	157.3	160.9	1.00	145.99	0.05	133.0	136.7	141.3	146.0	150.6	155.3	159.0
12	PS	0.93	148.34	0.05	135.5	139.5	143.7	148.3	153.0	157.2	161.3	0.88	147.88	0.05	134.1	138.5	142.9	147.9	152.8	157.3	161.8
	WHO	1.00	154.00	0.04	141.0	144.7	149.3	154.0	158.7	163.3	167.0	1.00	152.44	0.05	138.8	142.7	147.5	152.4	157.3	162.2	166.1
13	PS	1.02	151.85	0.05	138.8	143.0	147.2	151.8	156.5	160.7	164.8	0.97	154.22	0.05	140.1	144.6	149.1	154.2	159.3	163.9	168.4
	WHO	1.00	158.30	0.04	145.2	149.0	153.6	158.3	163.0	167.6	171.4	1.00	159.70	0.05	145.4	149.5	154.6	159.7	164.8	169.9	173.9
14	PS	1.09	154.16	0.04	141.1	145.3	149.5	154.2	158.8	163.0	167.1	1.04	159.77	0.05	145.3	149.9	154.6	159.8	164.9	169.6	174.1
	WHO	1.00	160.89	0.04	147.9	151.6	156.2	160.9	165.6	170.2	173.9	1.00	166.31	0.05	151.7	155.9	161.1	166.3	171.5	176.8	180.9
15	PS	1.13	155.79	0.04	142.7	146.9	151.1	155.8	160.5	164.6	168.8	1.10	163.92	0.05	149.4	154.0	158.7	163.9	169.1	173.8	178.3
	WHO	1.00	162.19	0.04	149.3	153.0	157.6	162.2	166.8	171.4	175.0	1.00	171.15	0.05	156.5	160.7	165.9	171.1	176.4	181.6	185.8
16	PS	1.17	157.13	0.04	144.0	148.2	152.4	157.1	161.8	166.0	170.1	1.14	166.77	0.05	152.2	156.8	161.6	166.8	172.0	176.6	181.2
	WHO	1.00	162.72	0.04	150.0	153.7	158.2	162.7	167.3	171.8	175.4	1.00	174.23	0.04	159.7	163.9	169.0	174.2	179.4	184.6	188.7
17	PS											1.17	168.73	0.05	154.1	158.8	163.5	168.7	173.9	178.6	183.1
	WHO											1.00	175.77	0.04	161.5	165.6	170.7	175.8	180.9	185.9	190.0
18	PS											1.19	170.25	0.05	155.6	160.3	165.0	170.2	175.4	180.1	184.6
	WHO											1.00	176.39	0.04	162.5	166.5	171.4	176.4	181.4	186.3	190.3

L, skewness; M, median; S, coefficient of variation.

^*^Statistically significant differences in height were found between the present study and WHO at all ages in both sexes, with a *p* value <0.001.

Finally, adult height and PHV observed at the 50th percentile (Mu) on the smoothed LMS curves were similar to the adult height estimated by the PB1 model, in both sexes, indicating an apparent agreement between the two methods.

## Discussion

This study is the first of its kind that presents height growth velocity and centile curves of Mexican boys aged 2 to 18 years and girls aged 2 to 16 years; among the main results were (a) Mexican boys and girls experience timing and tempo of height growth at TO and PHV at an earlier age; (b) the APHV was earlier for overweight-obese children than normal weight-for-age peers in both sexes; and (c) height values were different in the Mexican population as compared to the WHO’s reference values at all ages.

One of the most important findings was the early age at which PHV occurred. Girls reach PHV at 9.9 years and boys at 12.4 years. The timing of PHV in Mexican children was last reported in a population study conducted in the city of Mérida, Yucatán, in southern Mexico. Datta Banik et al. found that PHV in boys occurred at the age of 12.4 years, which is very similar to the result obtained in the current study. However, in girls, PHV occurred at the age of 11 years, which is 1 year later than that observed in the present study (3). On the other hand, Bogin et al. (38) reported the height growth of children of Mayan and Ladino descent (Spanish-speaking mestizo population) from Guatemala, a country with great similarities in physical and social-cultural environmental characteristics, and observed a PHV in Mayan girls and boys at 12.5 and 15.3 years, respectively, while, in Ladino girls and boys, it occurred at 12.0 and 13.6 years, respectively.

One population that presented a similar APHV to the present study is the Peruvian. A study carried out, between 2009 and 2010, in different regions of Peru showed an estimated APHV of 12.6 years in boys and 9.7 years in girls (39). In other populations, PHV has occurred at different ages. Chinese boys and girls assessed during 2008 reached PHV at 12.6 years and 10.6 years, respectively (40). Polish boys assessed in 2012 exhibited an APHV of 13.0 years (41); research conducted in Denmark showed an APHV of 13.8 years in boys and 12.2 years in girls for children born between 1996 and 2000 (42); in addition, Portuguese boys showed an estimated APHV of approximately 13.4 years between 2010 and 2015 (43). All the abovementioned studies fitted their cross-sectional growth data with the PB1 model.

Concerning other somatic maturation indicators, this study found a higher velocity at TO (5.24 cm/year among boys and 5.93 cm/year among girls) when compared to the results obtained in the studies representing Portugal (43), Peru (39), Guatemala (38), and even in the English population, which was evaluated almost 50 years before in the Harpenden Growth Study (4.51 to 4.9 cm/year) (33); however, the velocity at the age of PHV (6.87 cm/year in boys and 6.75 cm/year in girls) was similar to that of the Portuguese population (6.83 cm/year) and lower than that of Guatemala (9.63 cm/year), England (8.23 cm/year) (33), Poland (7.21 cm/year) (41), Denmark (10.21 cm/year) (42), and the South Korean population (8.62 cm/year) (44). The percentage of adult height at TO estimated in the present study was also lower (75%) compared to the aforementioned populations (76–79%), but the percentages of height growth from age at TO to PHV and from the PHV up to adult height (13.2 and 11.4%, respectively) were higher than those reported in most of the other studies mentioned above (<12.5 and < 10.9%, respectively).

Furthermore, the duration of the first stage of the adolescent growth spurt (age at PHV minus age at TO) observed in the present study in boys (3.76 years) was similar to that reported by other experts from Portugal, Peru, Guatemala, Denmark, and China, which ranged between 3.3 and 3.84 years and was similar to the Harpenden Growth Study (3.5 years) (33). Among girls, the duration of the first stage of adolescence (2.9 years) did not vary much with other studies, i.e., 2.6 and 2.9 years, in China (40) and the Harpenden Growth Study (33), respectively. However, the percentage of adult height at PHV was lower (87%) than the Peruvian (39), Chinese (40), and English (91–93%) (33) populations; consequently, greater height gain was reported from age at PHV to adult height (20.3 cm) than in the populations already mentioned (10.8 cm, 18 cm, and 15.1 cm, respectively).

Therefore, based on the analysis conducted in this study with a representative sample of Mexico, an earlier PHV was observed, with a more intense growth acceleration during the TO and less intense during the PHV, with a lower percentage of adult height during the TO compared to other populations, and a compensation in growth from age at TO to PHV and from the PHV up to adult height. In future studies, two important findings require attention: (1) confirming early maturation in the Mexican population based on other maturation indicators (such as menarche), as well as analyzing possible causes and consequences, and (2) studying the low growth during early childhood (until the OT) in comparison with other populations. Concerning the first issue, one of the main factors associated with early PHV in the Mexican population might be overweight and obesity. Currently, Mexico is one of the countries with the highest prevalence of childhood overweight and obesity (45); in this study, the prevalence of overweight and obesity in boys and girls showed age variations. In both sexes, overweight/obese was evident from 8 to 9 years until 16 years, with a prevalence higher than 30%. Numerous studies indicate that being overweight (measured by BMI), having high body fat, and experiencing rapid weight gain during childhood can lead to early bone, somatic, and sexual development, including early menarche, during childhood and adolescence. This association has been mentioned in several publications (46–51). However, Datta Banik (16) reported that higher adiposity and early maturity were also associated with short height. The author stated that earlier menarche results in a growth trade-off, leading to short stature, and that a bigger trunk relative to leg length, among girls, might increase the risk for body fat gain. Obese children have a positive energy balance that may affect the timing of maturation. Must et al. (52) observed that childhood obesity is “auxogenic” since the excess of calories (positive energy balance) leads the individuals to obesity and simultaneously increases their growth velocity. Earlier age at TO and PHV in Mexican girls can also be associated with the secular change of age at menarche since Mexican girls presented earlier age at menarche than their mothers and grandmothers (20, 53).

Although it is not yet possible to convincingly explain this physiological phenomenon, the association of earlier maturation with obesity, some hypotheses have been accepted as possible. Approximately 40 years ago, Frisch and Revell (54) suggested a critical weight (48 kg) for menarche to occur. This hypothesis was then severely criticized. However, the recent discovery of leptin confirmed Frish’s theory showing that adipose tissue is closely related to gonadotropin-releasing hormone activation, and fat mass can be responsible for the trigger of puberty (17). Moreover, body weight and fat gain can develop insulin resistance and beta cell dysfunction causing compensatory hyperinsulinemia and reducing the levels of sex hormones binding globulin (55, 56). All these aspects are possibly associated with height growth acceleration, thelarche, and early menarche in girls (57–59).

In the present study, while estimating the height growth characteristics of normal-weight and overweight-obese adolescents separately, a difference of 0.9 years was observed in the APHV between girls of adequate weight (10.37 years) and overweight and obese (9.47 years). However, boys showed a considerably smaller difference, 0.26 years (12.67 years in normal weight vs. 12.41 years among overweight-obese). Since boys lose more fat during early adolescence, the onset of the growth spurt and sexual maturity is associated with a smaller quantity of adipose tissue and total body fat, and, therefore, the timing and tempo of maturation can have less impact in boys with overweight and obesity conditions (60). A recent study carried out by Deardorff et al. in 2021 in a Mexican American population (61) found an earlier onset of puberty in overweight and obese girls (thelarche onset and early menarche) than in girls with adequate weight-for-age, whereas, in boys, the level of overweight and obesity was not related with earlier pubertal onset. Literature has shown to be more conclusive regarding the interrelationship between obesity and early onset of puberty in girls than in boys (62).

On the other hand, growth velocity in overweight boys and girls was higher at the age at TO and lower at APHV than in normal-weight boys and girls, presenting the obese subjects with an apparent compensatory growth effect (negative catch-up growth), similar to that presented by premature children who were born shorter and then increased somatic growth to catch-up growth potential (positive catch-up growth), or early weight gain and subsequent growth in leg length (63).

Furthermore, overweight and obesity prevalence varied in the Mexican regions as presented in the Results section. The percentages are mentioned under the assumption that the observed growth curve may be different among the different regions since Mexico is one of the countries with the largest territorial extension and remarkable physical, sociocultural, economic, and environmental differences. Given that the prevalence of overweight and obesity is almost double in the center and southern regions of the country compared to the north, and, apparently, overweight and obesity have an early effect on the timing of maturation, mainly among girls, it could be of great interest in the future, to carry out auxological studies based on specific geographical areas and environmental factors. Yokoha and Higuchi (64) observed that Japanese populations of different geographical areas reached the PHV at different ages, and this geographical difference was explained mainly by the rate of overweight. In addition, other factors that could affect the maturity process include altitude. A study from Colombia reported no significant differences in APHV, PHV, and estimated final height between the children and adolescents living at low and moderate altitudes (65). On the other hand, Peruvian children and adolescents living at different altitudes showed differential timing and tempo of growth; individuals living at sea level experienced an earlier APHV, were taller at APHV, had a higher PHV, and had a taller estimated final height compared to peers living at higher altitudes (39).

In other outcomes of the present study, height growth was lower than in other populations at infancy and early childhood (above mentioned), and height values were lower than those of the WHO values (on average 6–7 cm) at all ages in both sexes. Diverse factors, such as economic inequality, urbanization, migration from rural to urban regions, geographical and sociocultural differences, and food security, all together might have influenced the lifestyle habits of the Mexicans, resulting in these new conditions and a secular trend effect of children’s height (66–68). It was observed in Mexican children that stature can vary between those living in moderate and extreme poverty and those who are not poor (69). Socioeconomic status was observed to be associated with stunting conditions, mainly in Mexico, where approximately 40% of children with stunting are estimated to be in the low and very low socioeconomic levels (70). Furthermore, it has been observed that moderate-to-severe food insecurity, low birth weight, and low maternal height are factors associated with stunting (71). This study is the first step toward understanding the reality of somatic maturation in the Mexican population; however, future research on this topic could also analyze the impact of lifestyle and other environmental factors on the timing, tempo, and magnitude of biological maturation.

Furthermore, lower height was graphically represented by two kinds of curves: (1) the velocity curves based on the increases or the differences observed considering two consecutive moments of observation, built through the PB1 model and which represent the process of growth; (2) the distance curves, which are representative of the absolute value achieved in a specific moment and is the product of growth, generated in the present study by the LMS smoothed percentile growth curves.

Although the data used by the WHO to generate reference values are representative of the global diversity of children and are adjusted to internationally accepted health and growth standards, the Mexican population is not included; therefore, it is relevant to have reference values for the Mexican population, which may be a useful tool to complement the existing WHO values.

In this study, smooth percentile curves for height using the LMS method were created. However, it is important to note that these curves were not meant to be used as reference graphs for Mexico’s population. For that purpose, a more extensive statistical analysis would be necessary; however, they can realistically represent height growth by age and sex and can be used, in the future, for comparisons with other studies conducted in different Mexican populations. In addition, these results may be useful for monitoring the growth and development of Mexican children, identifying possible growth problems and improving medical care and child nutrition in Mexico. Moreover, the Lambda and Sigma values obtained in the percentile data of the Mexican population were similar to those observed by the WHO, which represented a smooth transformation and a low coefficient of variation. The values of bias and variation were similar to those observed by Kuczmarski (72) for the weight and height references of the US population.

One of the limitations of the present study was the use of cross-sectional data given the nature of the sample size and the type of sampling (multistage random); however, it will be convenient in the future to carry out longitudinal studies in the Mexican populations. Furthermore, growth was not modeled based on socioeconomic, geographical, or lifestyle habit factors; however, it is also an area of opportunity for future studies. Although the present study did not focus on sexual maturation, we acknowledge the importance of this aspect of development. Therefore, another future line of research should focus on the analysis of the stages of sexual maturation stages; this is particularly important in Mexico, where there is limited information on this topic.

In conclusion, we present the first report of height growth patterns, using the PB1 model and centile curve (LMS) fitted in a representative sample of 2-to-18-year-old boys and 2-to-16-year-old girls from Mexico. Mexican boys and girls experience an earlier TO and PHV, with a slightly low TO intensity, compensated during the growth spurt. Additionally, a distinct timing and tempo of the TO and PHV were observed when comparing normal-weight and overweight-obese boys and girls, being the study physiological events earlier and the growth velocity lower at TO and PHV in the overweight-obese group, in both sexes. Finally, the Mexican population presented a shorter stature compared to the WHO reference values at all ages in both sexes, which may correspond to a genetic condition or a stunting condition, which could not be identified in the present study. However, this will be an interesting topic to explore in future research.

## Data availability statement

Publicly available datasets were analyzed in this study. This data can be found at: https://www.inegi.org.mx/programas/ensanut/2018/#Datos_abiertos.

## Ethics statement

Ethical approval was not required for the study involving humans in accordance with the local legislation and institutional requirements. The studies were conducted in accordance with the local legislation and institutional requirements. Written informed consent to participate in this study was not required from the participants in accordance with the national legislation and the institutional requirements.

## Author contributions

LF: Conceptualization, Data curation, Formal analysis, Methodology, Writing – original draft, Writing – review & editing. SD: Formal analysis, Methodology, Validation, Writing – review & editing. NC: Supervision, Writing – review & editing, Formal analysis, Validation. IF: Supervision, Writing – review & editing, Conceptualization, Investigation, Methodology.

## Footnotes

^1^ https://www.inegi.org.mx/programas/ensanut/2018/#Datos_abiertos
